# Two Levels of Integrated Information Theory: From Autonomous Systems to Conscious Life

**DOI:** 10.3390/e26090761

**Published:** 2024-09-05

**Authors:** Zenan Ruan, Hengwei Li

**Affiliations:** 1 Department of Public Administration, Hangzhou Institute of Administration, Hangzhou 310024, China; znruan@zju.edu.cn; 2 School of Philosophy, Zhejiang University, Hangzhou 310058, China; 3 Center for the Study of Language and Cognition, Zhejiang University, Hangzhou 310058, China; 4 The State Key Lab of Brain-Machine Intelligence, Zhejiang University, Hangzhou 310058, China

**Keywords:** consciousness, Integrated Information Theory, theory of consciousness, life, necessary and sufficient mechanism

## Abstract

Integrated Information Theory (IIT) is one of the most prominent candidates for a theory of consciousness, although it has received much criticism for trying to live up to expectations. Based on the relevance of three issues generalized from the developments of IITs, we have summarized the main ideas of IIT into two levels. At the second level, IIT claims to be strictly anchoring consciousness, but the first level on which it is based is more about autonomous systems or systems that have reached some other critical complexity. In this paper, we argue that the clear gap between the two levels of explanation of IIT has led to these criticisms and that its panpsychist tendency plays a crucial role in this. We suggest that the problems of IIT are far from being “pseudoscience”, and by adding more necessary elements, when the first level is combined with the second level, IIT can genuinely move toward an appropriate theory of consciousness that can provide necessary and sufficient interpretations.

## 1. Introduction

Since Crick’s great initiative [1] and the introduction of the concept of neural correlates of consciousness (NCC), the study of consciousness has gradually moved from being shunned by empirical science to being accepted in the late 20th century [2]. Up to now, this field—science of consciousness—has developed a large number of theories of consciousness with varying viewpoints to date [3], and Integrated Information Theory (IIT) [4,5,6,7] is one of the most prominent candidates, which claims it starts from the phenomenology of consciousness itself and then proposes the conditions to be met for judging a physical system as the substrate of consciousness.

IIT is enduring many criticisms [8] in trying to live up to expectations, and in summary, the main challenges stem from three factors: (1) Phenomenologically, some have argued the properties of experience that IIT describes lack other components, such as a “point of view” [8,9], the conscious field [10], the function of agency [11,12], et al. (2) For the physical (operational) mechanisms, IIT’s theoretical (especially mathematical) framework [13,14,15] has been refuted, as well as its commitment to the neural implementation or the localization of consciousness in the brain [16,17,18]. (3) In fundamental metaphysics, IIT is bogged down in how it deals with panpsychism [19,20]. The tendency of panpsychism is arguably the central aspect of the challenges facing IIT. It is the “combination problem” (cf. [21]) of panpsychism that requires IIT to make further improvements in its principle of measurement, and in the case of conscious phenomena, IIT confuses certain subjective stuff or properties in the sense of panpsychism (e.g., proto-mentality) with consciousness, which leads it to make some unacceptable predictions (e.g., a diode may be conscious).

In this article, we argue why IIT has fallen into this trap and how to get out of it. Unlike others, we do not look merely for problems in the theoretical description of IIT, but we are particularly concerned with the positive development of its impressive quantitative principle. To do so, we must first understand which important results the IIT’s quantitative principle provides, and we have to reinterpret those results—what they can tell us in the most immediate sense. Comparing these substantial achievements with the complete framework that IIT claims to be using to explain consciousness, we can more clearly see the possible flaws and corresponding improvements in IIT.

The main work of IIT is reflected in an exquisite and complete measurement principle. Throughout IIT’s history, its principle has changed. Section 2 focuses on these changes during its development and summarizes several aspects of IIT’s effective work. In Section 3, we will discuss the meaning of IIT’s working at two different levels and respond to the gap between the two levels in Section 4.

## 2. The Three Issues in Historical Versions of IIT

The development of IIT has passed through several well-defined stages, from IIT 1.0 [4,22] to the current IIT 4.0 [7]. In particular, before the formal proposal of IIT, the advocate Tononi had participated in the early work of consciousness science with Edelman, the Nobel laureate, which obviously provided some basic clues for the emergence of IIT [23,24,25]. To a certain extent, we can also think of Tononi’s work at this stage as the version 0 of IIT or Pre-IIT.

By taking a closer look at the specific features of the conscious brain, Edelman has believed it has to be complex enough, and “something that is completely random is not complex, nor is something that is completely regular”, and thus “only something that appears to be both orderly and disorderly, regular and irregular, variant and invariant, constant and changing, stable and unstable deserves to be called complex. Biological systems, from cells to brains to organisms to societies, are therefore paradigmatic examples of complex organizations” [25] (p. 135). To take the co-existing constraints of functional integration and differentiation of the brain into account, Edelman and Tononi conceived the neural complexity (C_N_) index—for a nervous system, C_N_ is low when its components are completely independent or interdependent, and C_N_ is high when its components possess both local independence in lower dimensions (micro grains) and dependence in higher dimensions (macro grains). Correspondingly, they proposed *the Dynamic Core Hypothesis*, which states that conscious experience arises from a subset of functionally highly integrated and differentiated neurons, i.e., functional clusters in the brain. Brain regions such as this are not a fixed subset in space. Instead, dynamic cause-and-effect relationships are formed and changed continuously in the brain based on neural connections.

In their hypothesis, a certain complexity is understood as a question of consciousness and the “dealing with plethora” [25] (p. 111) of the brain. They argued that any neural process capable of producing conscious experience must have sufficient complexity. Numerically, C_N_ conceived at the time is simply the mean of the mutual information between all parts of the system—obviously greater than the minimum integrated information of subsequent IIT, and less than all of the additional information under the maximum entropy assumption—as a compromise between the two extremes.

Early IIT 1.0 [4] followed Edelman’s line of thought, also using *mutual information* between two subparts to define two quantities: “effective information” measures all of the additional information under the maximum entropy assumption, and “information integration” is the smallest of all possible effective information between all subparts (the “weakest” joint between subparts). That is why IIT 1.0 was once named “Information Integration Theory”—a theory that emphasized the complex relationship between the two co-existing tensions of “information” and “integration”—and it is also a theory that emphasizes “integration of information”.

It was in version 2.0 [5] that the theorists began to rename IIT as Integrated Information Theory (although its acronym remained the same), and the mutual information measure was replaced by relative entropy, also called Kullback–Leibler divergence (KLD). Compared with the local relations of a system that mutual information is intuitive to, the relative entropy is oriented to the change of the whole system, but the basic account and calculation of the theory have mostly stayed the same. In further versions, the mathematical method of measurement has been replaced in turn by Earth Mover’s Distance (EMD, IIT 3.0 [6]) and Intrinsic Difference (ID, IIT 4.0 [7,26]). In the process of comparing two probability distributions, the relative entropy ignores the states associated with the corresponding probability values, and the EMD selected for IIT 3.0 involves both the probability values and states. ID is related to the relative entropy, but it presents an internal perspective of the measurements of system information, rather than the extrinsic perspective of a channel designer.

It can be seen that the measurements of both information and integration are fundamentally homologous, which is also reflected in the fact that they use the same mathematical implement. The distinction is that, when calculating information, what needs to be taken into account is the maximum difference (obtained) that the system can produce after partitioning, which is reached when the system parts are completely independent (maximum entropy). The calculation of integration takes into account the minimum difference (at least to loss) of the system before and after the division. If information is “differences that make a difference” [27] (p. 84), then integration really measures “differences that avoid a difference”.

Besides the selected methods of calculation, IIT 3.0 greatly strengthened the principle for how to define the value of integrated information (i.e., Φ) and correspondingly, the development of identical relations in more detail. The element about identities is a significant aspect of IIT as a theory of consciousness. In theory, IIT 3.0 does not first point out the measurement of integrated information but instead tries to establish a philosophic–mathematical framework by reflecting the basic characteristics of consciousness, though its improved principle of measuring Φ still plays a key role in such a framework.

As of IIT 2.0, the advocates began to think about how specific informational structures relate to some content of the experience in a system where its integrated information is greater than zero. At this point, the question is not only whether a system possesses a positive integrated information. The further question is, what does the so-called integrated information express? Also, for example, is there a difference between two systems with the same value of integrated information, and how are they different? The strategy of IIT 2.0 to address these problems is to propose the concept of *qualia space* [5,28], which is the probability space in which the information structure of the system “resides”. In this way, the informational relationships within a physical system are mainly represented by some kind of hyperspatial geometry, and IIT 2.0 refers to such a geometry as the shape in qualia space. Take, for example, the classic “bat argument” [29], whereby once we know the physical state of the bat’s nervous system and its full causal mechanisms at a given moment, we can then calculate and acquire the geometry and position of its experience on the qualia space. In the later version 3.0, IIT has made a more detailed description and improvement on the above thought—the geometry corresponding to qualia has been transformed into the maximally irreducible conceptual structure corresponding to a constellation of concepts that are specified by the mechanisms of a complex (some part or subsystem of a given system), but the general idea remains similar [6,30].

With the measurements that had been applied during these historical versions, the IIT totally focuses on three issues as follows:

- The “complexity” issue: Measuring complexity—high or low.
- The “criticality” issue: Judging some criticality—yes or no—which is whether Φ>0 (to be conscious) for IIT, although subject to measurements. This is also where IIT is fundamentally a theory independent of the dynamic core hypothesis.
- The “identity” issue: Clarifying the identity between consciousness and the information or systematic structure within a physical substrate.

Unlike the purely mathematical or engineering task of measuring integrated information, characterizing informational relationships is a task that mainly concerns the “identity” issue of IITs. In the early stages, the “complexity” issue and the “criticality” issue have taken center stage in IITs’ accounts, focusing on the interpretation and calculation of both the suggestive properties of “(intrinsic) information” and “integration”. The “identity” issue was concretized and formalized since IIT 2.0 and took up more space in theoretical exposition—although the measurements have always been a significant part of the expanding interpretative framework.

Notably, the “identity” issue is an indispensable aspect in the sense of accounting for consciousness, but this aspect must be based on both the “complexity” issue and the “criticality” issue. If there is no critical threshold for the degree of integrated information in a system, it is meaningless and worthless to characterize the informational relationships in this system, because in this case, there would be no intrinsic information in a phenomenological sense (with the word “intrinsic”, IIT refers to how a system with a positive Φ can be like a whole and have a first-person perspective). It could also be said that the first two issues provide a methodological basis for how to implement the “identity” issue. In principle, IIT can replace or improve the measurement upon Φ with diverse methods. However, its current formalization does display a meaningful grasp, which most of the negative comments that IIT has faced have largely not denied, although it is still continuously being optimized.

## 3. Two Levels of Integrated Information Theory

On the grounds of their relevance to the three issues generalized from the development of IIT, we have summarized the main ideas of IIT into two levels. At the first level, we focus on the quantitative advantages of it, and we need to articulate what this piece of elaborate computation actually says. At the second level, we notice that IIT proposes an interpretative framework of consciousness compatible with its quantitative principle, and we need to explore how the leap from the quantitative principle to interpretative frameworks could be achieved.

### 3.1. The First Level

For a theory of consciousness, the ability to judge whether a system is conscious or not, i.e., a response to the “criticality” issue, is the first requirement. This is why IIT has currently become one of the most prominent theories of consciousness: its principle of measurement and then judgment contains a criterion that is based on rigorous mathematical measurements compared to other theories of consciousness. Global Neural Workspace Theory (GNWT) [31,32] and Recurrent Processing Theory (RPT) [33,34], for example, both emphasize the unification of information brought about by reentry (or recurrence) among various brain modules. However, they do not provide a quantitative explanation of the degree to which information unification fulfills their requirements (global access for GNWT, formation of recurrent processing for RPT).

Although IIT 3.0 has developed a series of sophisticated calculations to create a rigorous judgment principle, it is still constantly being improved and developed due to some possible criticisms of details of the calculation. Not only does IIT use different methods to calculate the distance between two probability distributions at its different stages (as discussed above), but their measurement processes also vary. However, the principle given by IIT always revolves around the core idea of how to define an integrated system in terms of information operations, and the mathematical details are just optimized to make these operations more rigorous or more computable. With a better understanding of the context of the “criticality” issue, we can selectively ignore the ever-changing additional computational details while grasping the core idea of IIT measurements. Specifically, we want to know how the IIT’s calculations define how a system made of its parts has a distinct character that marks it off from a combination of its parts, that is, it cannot be reduced to its parts, i.e., *irreducibility* [35,36,37].

We illustrate this with some simplified cases that follow the specifications of any system defined by IIT. Let us start with a brief introduction to the basic definition of IIT measurement. Overall, IIT supposes that anything that is physically assembled is a system (at least in the aggregate sense) made up of elements, and these elements all form causal constraints according to their own logical functions and the functional connections between them. To facilitate the expression of the function, it is assumed that each element has a limited repertoire of outputs (the distribution of 0/1 in binary logic systems, such as synaptic activation or suppression, circuit logic gates on or off). Depending on their combination, the functional elements can function as different mechanisms in a system. For example, a system with n elements can have a total of $2^{n}-1$ possible mechanisms (except for the empty set).

For such a system model, IIT considers each of its parts (subsystems) as candidate systems—although a system itself may not be integrated, some of its parts can be integrated. On the other hand, even if there are several integrated parts, the most integrated part will dominate (exclusivity), so all candidate systems need to be measured accordingly, and of course the overall system is included in the candidate systems. By comparing the minimum amount of information that these candidate systems would lose after eliminating the causal correlation (by partitioning) between any two mechanisms, IIT selects as the “winner” the one that loses the least amount of information and can be labelled as a complex. Thus, the minimum amount of information that the selected subsystem loses through partitioning is the integrated information of the original system. In the total process of measurement, determining whether there is no partition in a subsystem through which it would not lose information is arguably the most critical step. In fact, when exclusion was not proposed in the version of IIT 1.0–2.0, a subsystem can be said to be complex as long as the minimum amount of information it can lose is greater than zero.

The connections between the functional elements imply that there may be a causality, which for IIT is the cause–effect power of the current states of the system on the possible states from the past and future. In its computational process, distributions of states in both the past and the future are described by the probability distribution of a particular set of states. After a system is divided, its past and future probability distributions may change (if the system is not integrated, there is at least one partition that does not change its past and future probability distributions). To determine whether and to what extent the probability distributions have changed, we need to calculate the “distance” between the probability distributions before and after the cut—the so-called integrated information is mathematically the value of the distance between the two probability distributions.

**Figure 1:**
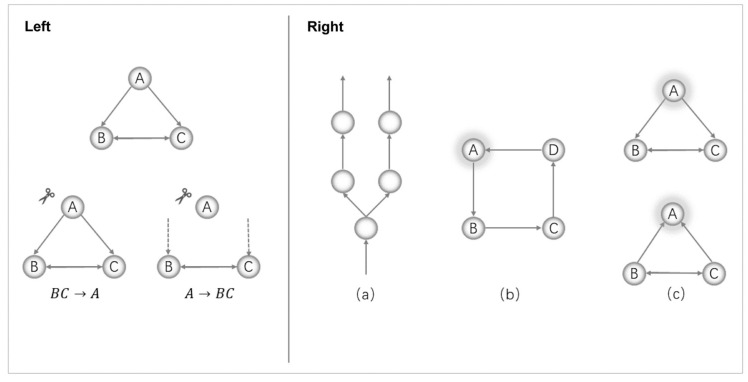
Illustration of an integrated system with irreducibility. For brevity, the specific mechanisms and the current status of the elements are omitted and only the directed connections of information transfer between the elements are considered. By default, the existing connection is distinct from the chance and noise. **Left**: The directed connection A→BC of the system ABC contributes a causal constraint to the system. Contrastively, the directed connection BC→A of the system ABC does not contribute any causal constraints, and the partitioned system ABC can therefore be reduced along the cut in this direction. **Right**: The system (**a**) consists simply of feedforward connections, in which each stage of information processing only influences the next layer and has no influence on the previous stage, and they do not form “one” system. In the system of example (**b**), element A is only directly connected to B and D, but there is also information sharing between A and C, and the current state of A would practically influence C and constrain the possibility of C in the past, and vice versa. In the case of the two systems in example (**c**), the element A either does not produce a constraint on the future of the remainder or does not constrain the possibility of the past of the remainder.

The mathematical processing of IIT assumes that a system *S* consisting of *n* interacting elements is subject to a Markov process, that is, the state of each element at ${time}_{t+1}$ depends only on the state of the system at ${time}_{t}$ and is not restricted to the state of the other elements at ${time}_{t+1}$ (with the exception of instantaneous causality, where each element is conditionally independent at the same time). A discrete dynamic Markov system can be described by a directed graph with interconnected nodes, each of which corresponds to a function that, given the state of its parent at ${time}_{t}$, determines the state of the node at the next step ${time}_{t+1}$. In simplified diagrams, as Figure 1 shows, we assume that each element has a function that is distinct from chance or noise. For brevity, however, we ignore the specific function trajectories, which would not detract from our purpose of showing exactly what integration requires in this directed graph.

If in an integrated system, the connection between any mechanisms is cut with a change in the probability distribution of the system, then the interdependence between the two partitioned mechanisms is no longer valid. In this case, the causal structure, still considering it as “one” system, must be completely different from the causal structure of the whole circuit before partitioning. 

Just as water flows always converge to the lowest terrain, the integrated information of a system should depend on the partition that causes the least causal change. In a system, there should certainly be partitions that can cause change, but if there is a partition where the joint probability distribution of the partitioned parts of the system matches the probability distribution of the original system before partitioning, then its integrated information would be zero—it is not integrated, because at least along that partition it can be reduced to two separate parts without any change in causal properties. In the partition example on the left of Figure 1, the directed connection BC→A of the system ABC does not contribute any causal constraints, and the partitioned system ABC can therefore be reduced along the cut in this direction.

The right of Figure 1 shows three examples of systems with different connected relations, and only the network (b) is irreducible. This irreducible wholeness actually results from the “interlocking” of causal constraints between the parts of a system, an organizational property that Hartshorne requires for the enduring individual [38]. Etymologically, the word “individual” corresponds to “indivisible”, which is irreducible and has no real parts—no other individuals as parts within it.

IIT 2.0 has used a similar concept, “entanglement” [5], in its description of intrinsic information. Fully interlocked or entangled would mean that each element of the system can influence each of the other elements, not just those that physically feed it information directly and receive its output. The system (a) on the right of Figure 1 consists simply of feedforward connections. There are a large number of such connections in the computer, just like most neuronal structures in the cerebellum [27] (pp. 56–58). In this cascade processing, each stage of information processing only influences the next layer and has no influence on the previous stage. Strictly speaking, they do not form “one” system together. In contrast, the integrated system requires a way of feedback processing, and the feedback of information in the network structure makes the causal relationships between mechanisms denser. In the system of example (b), element A is only directly connected to B and D, but there is also information sharing between A and C (A–C is bidirectionally “information through”), and the current state of A would practically influence C and constrain the possibility of C in the past, and vice versa. In the case of the two systems in example (c), element A either does not produce a constraint on the future of the remainder or does not constrain the possibility of the past of the remainder.

From the concise analysis above, we can see that the essence of “integrated information” in the principle of IIT is the “irreducibility” of the system, which possesses that extra intrinsic information than its parts, and we point out that this is the most concise conclusion of the first level of IIT.

The first level is important because it picks out some important system properties. Even in this sense, IIT should be enough to be a valuable scientific theory. The relevance of IIT, at this level, to the “identity” issue is relatively weak, and this is mainly involved in versions 0–2.0. Then, a system could be conscious as long as it had non-zero integrated information. However, we tend to understand IIT at this level not so much as a theory of consciousness, but as an information theory of “integrated information” itself, and particularly, the continuing work of Hoel [39] and others, some of whom were members of Tononi’s group, on causal emergence is the advancement of IIT at this level [40,41,42,43,44,45].

### 3.2. The Second Level

IIT 3.0 has been a milestone in the development of IIT, and it provided an exquisite framework for associating specific information structures with features of consciousness, which we summarized in Table 1. 

According to IIT 3.0, judging an experience is equivalent to confirming a maximally irreducible conceptual structure (according to Φ), and judging a concept is equivalent to confirming a maximally irreducible cause–effect (according to φ), in which an experience should consist of concepts. The experience and concept are considered, respectively, at two levels of system granularity. When looking for a concept, we use a single mechanism (including first-order and higher-order mechanisms) as a specific object of measurement. When we look for an experience, we take a candidate subsystem (consisting of multiple mechanisms) as a specific object, in which any mechanism within it now plays the role of an elementary unit. As we show in Table 1 for a case of a system, every mechanism with a φ^max^ specifies a *concept*, and it is the greatest irreducible conceptual structure (with a Φ^max^) of the set of concepts that uniquely specifies that particular (intrinsic) *experience*.

Through Table 1, we try to show that the identity relationship within IIT does not exist deservedly but is based on a set of narrative assumptions and measurement principles. Concretely, within the framework, a set of systematized identical relations for interpreting consciousness with five pairs of axioms–postulates (existence, composition, information, integration, and exclusion) is explicitly presented for the first time. Its existence was recently replaced by intrinsicality in IIT 4.0, but the basic purpose and account remain unchanged. For IIT, there can theoretically be no experience in the entire universe that does not fulfill these five axioms, and the corresponding five postulates with the same name describe the properties of a physical system that can produce such an experience: if a system is judged as capable of experience, it must possess these five properties. Meanwhile, information and integration are not only the first of these properties but also the most important aspects related to the computational analysis of measurements (exclusion also plays a role in the final judgment), and the rest are more simply proposed and improved to enhance the ongoing theoretical explanatory power.

To avoid possible misunderstandings, IIT 4.0 reversed the explanatory path of IIT 3.0 with a sophisticated measurement [46] that blends *concepts* into *experience* (a conceptual structure). However, IIT 4.0, as well as IIT 3.0, no longer examines the two aspects of “information” and “integration” separately like the early versions did but combines them in the computational process of the two dimensions of a single mechanism and system (multi-mechanism) (see Table 2).

At the second level of IIT, the identity relationship between information structures and subjective phenomena, which the “identity” issue concerns, becomes more concrete and occupies more and more space in the theoretical statement. However, Table 1 shows how this identity relationship is essentially mediated by a judgment of some complexity. In the most rigorous case, judging the complexity of a certain information structure is enough to establish a theory (i.e., the first level of IIT). Using our imagination, we can use this complexity as a mediator to orient the information system toward other concepts (for example, we think autonomy and agent can be more appropriate). To the unacceptability of many critics, at the second level, IIT oriented it toward consciousness, which in many ways remains vague and subject to much controversy. In our opinion, the account of IIT for consciousness only superficially gives narrative evidence. Some readers may not delve into how the identities are implemented, and when they are deconstructed, the problem becomes apparent. Although IIT is a theory dedicated to consciousness per se, the mediator of information complexity should be its most scientifically valuable part.

In the thinking before IIT 3.0, the description of the condition of consciousness was succinct, requiring only the judgment of whether a system’s integrated information has passed the critical point. In this way, the transition from the “criticality” issue to the “identity” issue has a direct given assertion, which seems to lack sufficient evidence. After version 3.0, IIT provided an axiom–hypothesis framework to fill in the interpretation of this leap. But is this addition reasonable enough? In fact, much of the criticism has focused on this part of IIT. Just dealing with the “complexity” issue and the “criticality” issue is a relatively sound part, and it is this part that gives IIT a quantifiable method to stand out from other theories of consciousness.

## 4. Bridge the Gap—IIT Needs More Elements

As discussed above, IIT describes some elements of consciousness at the first level, but it still needs to be completed. In terms of the conditions under which consciousness arises, some complexity is likely to be an important and unique necessary condition, but not a decisive one—the critical complexity of the existence of a system as a whole may indicate that it is an autonomous subject but not necessarily conscious. In this sense, IIT serves as a theory of consciousness that provides some necessary mechanisms for how consciousness arises. However, it is not yet a theory capable of providing a necessary and sufficient model of consciousness. By contrast, IIT has stepped up to give a far-fetched proposition at the second level, packaging a necessary theory of consciousness into a necessary and sufficient theory with certain narrative grounds (especially those identities in Table 1).

### 4.1. Elements in the Condition “Producing” Consciousness

The logical concepts of necessity or sufficiency often appear in the discussion of the conditions for the emergence of consciousness. However, as Lau [47] noted, while the logic of necessity or sufficiency is simple in itself, analyzing them in relation to a particular issue requires vigilance. For example, the operation of the engine system may be the main cause of a car moving, and the transmission system also plays a role in driving, but if the transmission system fails, the car can continue to drive without being able to shift gears. In addition, there are many options for the engine system. The car can be driven by a conventional engine, it can be driven by an electric motor, and it can even be pushed by a human hand to move the car.

As far as the terms are concerned, sufficiency expresses more additional limitations and thus restricts a narrower range of things. In comparison, necessity involves fewer restrictions and, therefore, restricts a wider range. For example, under the premise of biological consciousness, living is a condition with fewer restrictions that all living things actually fulfill and is obviously only a necessary condition for consciousness. Instead, the set of characteristics that includes the ability of “rational decision-making” should be a sufficient condition for consciousness. If we do not invoke the higher rational function, we can still have conscious experiences.

In the evolution of consciousness, its necessary conditions emerged first. As organisms evolve toward higher degrees of complexity, higher functions accompanied by more qualifications, such as the transition from “living” to a set of characteristics that are “not only living but have a nervous system” gradually developed. The mechanisms of “minimal consciousness” that we mentioned were the starting point, which is just enough to generate consciousness as the biological structure continues to become more complex and the restrictions of the necessary basic conditions continue to increase, corresponding to a necessary and sufficient condition for consciousness [48].

**Figure 2:**
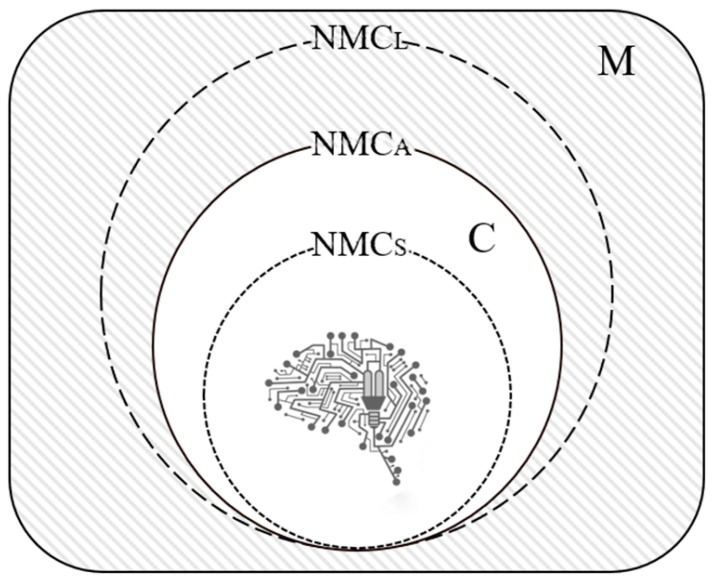
The logic of the identification of consciousness. The neural mechanism of consciousness (NMC) should represent a model of necessary and sufficient theory in the identification of consciousness with operational conditions, i.e., the NMCs are appropriate, labeled NMC_A_. NMC_S_ refers to a theory that is sufficient but unnecessary for the identification of consciousness, i.e., it excludes some of the neural mechanisms of consciousness and results in a small theoretical scope. NMC_L_ refers to a theory that is necessary but insufficient for the identification of consciousness, i.e., it includes content that is not part of the neural mechanism of consciousness, resulting in a theory with a large scope. Strictly speaking, only NMC_A_ can accurately match consciousness (C) in all properties of the mind (M).

The essence of the test of empirical theories of consciousness is that the model of a theory, specifying the mechanisms of consciousness, should be both necessary and sufficient to solve the problems of consciousness. The ability to provide a necessary and sufficient neural mechanism to account for consciousness is an important principle in determining whether a theory of consciousness is “appropriate” (see Figure 2). The range of mechanisms a theory gives can be neither “too narrow” nor “over-broad”. Because the “too narrow” mechanism could misidentify the actual conscious state as unconscious, such a mechanism is sufficient, but not necessary. The “over-broad” mechanism that falsely identifies the unconscious state as conscious is necessary but not sufficient. Only the “appropriate” theory can make a correct identification with consciousness, and the neural mechanism of consciousness it provides is thus necessary and sufficient to determine whether a system and a certain state of the system are conscious.

### 4.2. A Perspective of Life

Like some others, we think that looking at IIT and consciousness from a biological perspective can be instructive and helpful.

The first understanding and knowledge of consciousness came from conscious humans themselves, and a human belongs to the biological realm. A generally accepted common sense view is that consciousness did not originate with the most primitive life but only when life had evolved to a certain stage. Therefore, exploring the origin and nature of consciousness from the perspective of life and evolution has to be a natural entry point to the study of consciousness.

Evolution refers to the physiological changes and development of biological species over time, which is not only the evolution of physical structure, but also the evolution of the corresponding mental characteristics. However, although most scientists and philosophers today are largely convinced that consciousness is a biological process and a product of evolution, its evolutionary origins have rarely been the focus of discussions in its theories. Ginsburg and Jablonka [49] have pointed out that evolution is the most universal framework for understanding the biological world that any theory of life and also of mind must face, and that if these theories are not well compatible with it, there must be some problems in these theories that need to be fixed.

In Damasio’s system [50], the biological mind has evolved from nothingness, which has only a basic life that keeps itself in balance, to the nonconscious mind, to the conscious mind, and even further to the reflective mind. Nonconscious activity differs from unconscious mental activity in that the former means that the living being possessing the mind has no conscious basis at all, while the latter unconscious activity is simply not yet conscious and can become conscious at any time, that is, the owner of the mind already has the basis for consciousness. Damasio believes that consciousness arises from a mixture of inner and outer sensations, with the inner sensations being the more crucial facilitator, and that “each homeostatic feeling is itself spontaneously and automatically conscious” [51]. In particular, he argued that the nervous system alone cannot provide a response to the mechanism of consciousness, and that being conscious is an internal cooperative state that connects the nervous system and the mental processes it generates with non-neural entities, simply the body or the regulating parts of the internal organs.

In this sense, consciousness is a “product” of the most recent evolution of organisms and is based on the essential maintenance of life. Thus, a crucial step in the study of consciousness can be the search for “minimal consciousness” [52], or in other words, the search for certain markers and conditions that show that consciousness has just begun to establish itself. This particular transition becomes a natural barrier in the evolutionary history, a temporal criticality in which the organisms before are simply life without any inner experience and the organisms after, although seemingly not very different from those before, have dramatically altered inner worlds. For a causally closed network of electronic components, a change in the state of any one unit will affect the probability distribution of the entire network, which, according to IIT, has positively integrated information, thus being on a unique side when facing a judgment of some requirement of criticality. However, based on common sense, it would strike many as absurd that this network is not even a living one. The current IIT places the boundary of “minimal consciousness” in the smallest detail of the universe outside of the world of life.

### 4.3. IIT Needs More Elements

From the biotic perspective, the principle of whether a system is integrated is clearly not the necessary and sufficient condition for consciousness [48], and at least, no property of mind would be possible without the fundamental necessity of being alive. The panpsychist tendencies of IIT expose the fact that it is detached from the perspective of life, and it is this underlying fact that leads to many seemingly absurd conclusions.

We have to point out the two levels involved in IIT’s general framework, and we argue that there is a clear gap between these two levels in the theoretical interpretation of IIT for consciousness. Corresponding to this is the two asymmetrical facts. For one thing, the systems it measures are not limited to living organisms but to general information systems that are widespread throughout the universe. For another, IIT treats the posterior “hot zone” [53] in brains as the physical areas where consciousness can arise, particularly as a typical representative of the “posterior theory” of consciousness against with the “anterior theory” or “global theory” represented by theories such as GNWT. In these latter contexts, IIT is more like a neuroscientific theory of consciousness. 

In the context of neuroscience, the “criticality” issue, while important, is by no means decisive, and this was the thinking behind pre-IIT. In the *dynamic core hypothesis*, the study of the index of neural complexity is only one of the dimensions of consciousness to emerge, and this dimension obviously is closely related to other brain activities. At the first level of IIT, it corresponds to this idea that there may be no direct identity relationship between consciousness and the information structure. Theories at this level involve only the “complexity” issue and the “criticality” issue and can be used as a “pre-theory” of consciousness.

At the first level, IIT should not be a theory of the direct anchoring of consciousness. Based on the explanatory framework of IIT, consisting of five axioms–postulates and its emphasis on the existence of the integrated system as an irreducible “whole”, one could think of the concept of the *monad* proposed by Leibniz [54]. The emphasis on irreducibility is a significant change in the “criticality” issue of IIT 3.0. How can a system (subsystem) become a whole? This problem was contained within the “complexity” issue according to the dynamic core hypothesis of pre-IIT, and further versions of IIT began to require that there is a definite boundary or threshold. IIT demands that a whole must be indivisible—any division would inevitably lead to a change in its information content. However, this causal constraint is not dense during IIT 1.0–2.0, with which the measurements only consider the effect of the current state on the probability distribution of the system in future, so that a mere unidirectional connection between two elements may produce a positive Φ [4,5]. IIT 3.0 considers bidirectional probabilistic transitions of both cause and effect (past and future) and focuses on the cause–effect information, not just effective information. Therefore, the complex, defined by IIT 3.0, has considerable solid density, which as a whole is completely impossible to reduce in any direction to a combination of its parts. The theorists also think of such a whole as self-generated, self-referential and holistic [6].

Similarly, the *monad* is the smallest unit as an entity, which, in the sense of the “whole”, does not have any components. Each *monad* that has its own rich internal world is entirely defined by its own world and operates according to its own past and future intrinsic causal power, thus having a unique subjective view of the external universe. Without its religious overtones, such a concept of being is a good description of any autonomous agent that exists as itself, the subject. Any subject is defined by itself, while the object, on the other hand, is only recognized by things outside and thus defined and described. In the context of life and evolution, autonomy is a requirement far below the capacity to be conscious. Autonomy is not identical to free will. Free will is a higher requirement of living organisms, and even humans, in infancy, probably do not have sufficient free will—free will is obviously a higher requirement than consciousness. The *monad* has some primitive subjectivity, but it is not a living organism and cannot possibly produce consciousness, and the same is true of the complex judged by IIT.

Nonetheless at the second level, the measure of complexity has assumed the sole, primary ontological role in the existence of consciousness. In this case, IIT provides an account for some of the information complexity present in the system structures that give rise to conscious experience. This level includes not only the problems of degree required by the “complexity” issue and then the “criticality” issue but also the informational relationships required by the “identity” issue. 

The IIT’s overly strict identity makes it possible for non-brain, inanimate systems to possess consciousness. At the first level of IIT, the measurement and judgment are based merely on the mathematical characteristics of the system, while at the second level, the other side of the identity introduces entirely different properties of life. We see that there is a clear “leap” between the criticality of complexity and the identities between information structures and subjective phenomena, and it is this “dubious leap” that creates a gap between the two levels of IIT. Theoretically, IIT does not have to use current measurement principle as a basis for the identity; for example, we can also resort to the mechanisms of recurrent processing and others to conform to the axioms of the properties of consciousness.

So how can IIT close the gap in its theory? We believe that the current explanatory framework of IIT potentially contains two conflated objects, and therefore consists of two approaches that do not exactly overlap. One is dedicated to the explanation of an autonomous agent or subject, metaphysically like a *monad*, and the other is concerned with consciousness or experience—the former needs to be adapted to truly fit into the latter. IIT currently claims to have reached what we call the second level (oriented to consciousness), but in our view, it should not have reached this level. Its first level specifies a certain complexity; at that level, it is an excellent theory.

From a common sense point of view, we only feel the results of consciousness from living things in reality, so it is very likely that life is an additional factor IIT needs to consider. Our point is that it would be arbitrary to assume that the characteristics of consciousness exclude such properties as life from the five axioms, although, of course, it could be other, more specific and detailed mechanisms, than just the also vague concept of life.

The reason why IIT focuses on the “hot zone” depends on the current context of neuroscience experiments, which really does not mean that IIT can only be a brain theory of consciousness. Based on the existing IIT, an independent system with Φ greater than zero can be conscious outside of a human body. However, IIT could avoid much of the criticism it has received if it confined itself to a neuroscience theory. IIT certainly does not have to be just a neuroscience theory, and we propose this in an attempt to show that by moderately adding more constraints to its judging a system to be conscious can indeed make current IIT more convincing.

From its two disconnected levels, current IIT can provide a partial description of the conditions necessary for the emergence of consciousness—the neural networks of brain regions that maintain consciousness are necessarily of high complexity, but the neural networks of some complexity are not always conscious (insufficient). IIT still has to face the challenge on its way to a theory that provides the necessary and sufficient conditions for consciousness. However, since the judgment principle of current IIT only applies to general information systems, it needs to be further adapted. Judgment about the informality of systems alone cannot be sufficient to answer this question, as it requires an expansion of the various dimensions of consciousness. There must be many more dimensions involved in the emergence of consciousness. At least, the physics [55,56,57,58] and especially quantum physics [59,60,61] might play a fundamental role in judging an empirical biological (physical) system. Formally, further elements need to be added to narrow down a defined set of constraints, e.g., by replacing an integrated system with an integrated living organization that meets the requirements of judgment in several dimensions. Of course, in the end, a reasonable project definitely needs more involvement in the argumentation of various dimensions of consciousness, and some researchers have made preliminary attempts [62,63,64].

## 5. Conclusions

IIT has retained two relatively separable parts in its ongoing development. The first level of IIT is mainly about the quantitative advantages of it, which provide a principle of measurement to judge if any system is complex enough to achieve some criticality. The second level aims at the identities between informational structures and subjective properties that are based on the judgment of critical complexity. 

We adopt a biotic perspective to inspect IIT and consciousness, from which a “dubious leap” between IIT’s two levels is presented. At the second level, IIT claims to be strictly anchoring consciousness, but the first level on which it is based is more about autonomous systems or systems that have reached some other critical complexity. Thus, the reason why IIT, as a prominent candidate for a theory of consciousness, has received a lot of criticism is mainly because there is a clear gap between its two levels of explanation, and its panpsychism tendency plays a crucial role in this.

Generally, the problems of IIT are far from being “pseudoscience”. It provides excellent scientific work on mathematical measurements at its first level, although it still has room for improvement on statements that combine the two levels. By adding more necessary elements, we believe that when the first level is combined with the second level, IIT can truly move toward an appropriate theory of consciousness that can provide necessary and sufficient interpretations.

## Figures and Tables

**Table 1:** The identities between information and phenomenal characteristics of a physical system.

Intrinsic experience	Maximally irreducible conceptual (cause–effect) structure	With Φ^max^	Other concepts			Complex (a subsystem)	System	
		Conceptual (cause–effect) structure						
			Concept	Maximally irreducible cause–effect	With φ^max^			
					Mechanism			
outside of experience								

* The gray background indicates the phenomenal characteristics.

**Table 2:** The basic measurement frameworks of IIT 2.0 and IIT 4.0.

	IIT 2.0	IIT 3.0	IIT 4.0	Phenomenology
information	effective information	integrated information structure	system integrated information	underlying richness
integration	integrated information			the degrees of experience
informational relationships	a shape in qualia space	a constellation of concepts	a Φ-structure	the content of experience

## Data Availability

The original contributions presented in the study are included in the article, further inquiries can be directed to the corresponding author.
